# Different Surgical Approaches for Early-Stage Ovarian Cancer Staging. A Large Monocentric Experience

**DOI:** 10.3389/fmed.2022.880681

**Published:** 2022-04-25

**Authors:** Stefano Cianci, Vito Andrea Capozzi, Andrea Rosati, Valerio Rumolo, Giacomo Corrado, Stefano Uccella, Salvatore Gueli Alletti, Matteo Riccò, Anna Fagotti, Giovanni Scambia, Francesco Cosentino

**Affiliations:** ^1^Unit of Gynecology, Department of Human Pathology of Adult and Childood ‘G. Barresi’, University of Messina, Messina, Italy; ^2^Department of Medicine and Surgery, University Hospital of Parma, Parma, Italy; ^3^Dipartimento Scienze Della Salute Della Donna e del Bambino, Fondazione Policlinico Universitario A. Gemelli IRCCS, Rome, Italy; ^4^Department of Obstetrics and Gynecology, AOUI Verona, University of Verona, Verona, Italy; ^5^Department of Public Health, Service for Health and Safety in the Workplace, Reggio Emilia, Italy; ^6^Department of Medicine and Health Science, “V.Tiberio” Università Degli Studi del Molise, Campobasso, Italy; ^7^Department of Gynecologic Onocology, Gemelli Molise SpA, Campobasso, Italy

**Keywords:** laparoscopy, robotic, laparotomy, ovarian cancer (OC), early-stage

## Abstract

**Introduction:**

Ovarian cancer is the third most frequent gynecological cancer. In early stage ovarian cancer (ESOC) comprehensive surgical staging is recommended. Surgical staging is traditionally approached by laparotomy, although minimally invasive surgery can be a valid alternative in selected patients. This study aims to analyze the surgical and oncological outcomes of three different surgical approaches in a large series of patients.

**Methods:**

We retrospectively included all histologically proven ESOC cases treated between January 2014 and December 2017. ESOC was defined as stage IA to IIB according to the 2018 FIGO staging system. Subjects were divided into groups 1, 2, and 3, based on the surgical approach (open abdominal, laparoscopic, or robotic, respectively).

**Results:**

Within patients enrolled during the study period, 455 met the inclusion criteria. No difference in intraoperative complications was recorded in the three groups (*p* = 0.709). Conversely, a significant difference occurred in postoperative complications (16.2 vs. 3.8 vs. 11.1%, in groups 1, 2, and 3 respectively, *p* = 0.004). No difference was found in overall survival (OS) (32 vs. 31 vs. 25 months, *p* = 0.481) and disease-free survival (DFS) (26 vs. 29 vs. 24 months, *p* = 0.178) in groups 1, 2, and 3, respectively. At univariate analysis FIGO stage I (*p* = 0.004) showed a lower recurrence rate compared to FIGO stage II.

**Conclusion:**

No significant difference was found in OS and DFS among the three groups (open, laparoscopic, and robotic). The minimally invasive approach showed lower rate of complications than the laparotomic approach.

## Introduction

Ovarian cancer (OC) is the third most frequent gynecological cancer worldwide (1). More than 70% of patients are diagnosed at an advanced stage because of the disease aggressiveness and the absence of early symptoms and adequate screening (2, 3).

The diagnosis of early-stage ovarian cancer (ESOC) (stage I-II disease) is usually incidental and associated with better survival, compared to advanced stages (4). In patients with ESOC, a radical surgical staging (RSS) including total abdominal hysterectomy, bilateral salpingo-oophorectomy, systematic pelvic and para-aortic lymphadenectomy, and radical omentectomy is recommended (4). RSS is traditionally performed by large midline laparotomies; however, due to the progressive technological improvements, minimally invasive surgery (MIS) has been increasingly adopted in the setting of ESOC (5, 6). Different studies showed that the MIS approach is associated with reduced hospitalization, fewer intra and post-operative complications, better cosmetic results, and superimposable oncological outcomes when compared to open surgery (7–12).

Given the rarity of ESOC, only few studies comparing laparotomy, laparoscopy, and robotics are currently available in the literature (13, 14). These studies have the major bias represented by small sample size, inadequate follow-up, and wide patient heterogeneity thus reducing the generalizability of the reported results.

The present study aims to analyze the surgical and oncological outcomes of the three different surgical approaches (open abdominal, laparoscopic, and robotic) for ESOC treatment in a large series of patients with a long-term follow-up.

## Materials and Methods

This is a retrospective monocentric study conducted at the “Dipartimento Scienze della Salute della Donna e del Bambino, Fondazione Policlinico Universitario A. Gemelli IRCCS, Roma, Italy” between January 2014 and December 2017. The IRB n. CICOG-31-10-18/212 was obtained. All patients provided written informed consent for their data to be collected and analyzed for scientific purposes. Data were extracted from the Research Electronic Data Capture (REDcap) database (Vanderbilt University in Nashville, Tennessee, USA) (15).

All ESOC cases, from IA to IIB International Federation of Gynecology and Obstetrics (FIGO) stage 2018 (2), were included. Age, FIGO stage, histologic subtype, American Society of Anesthesiologists (ASA) performance status, intra and postoperative complications, operative time, rate of conversion to standard laparoscopy or laparotomy, hospital stay, disease-free survival (DFS), overall survival (OS), recurrence rate, and time to chemotherapy, were collected for all patients. Histological slides were evaluated by dedicated pathologists with an extensive background in ovarian malignancies. The surgical approach was chosen based on patient BMI, previous surgery, ovarian lesion diameter, surgeon skill, and preoperative apparent FIGO stage. In the case of MIS approach, a laparoscopic endobag was used for ovarian lesion removal avoiding abdominal tumor spillage. Postoperative complications were categorized according to the Clavien-Dindo classification (16). Operative time was recorded from skin incision to skin closure. In the robotic group, the docking time was excluded. DFS was considered from the date of the histological diagnosis to the date of recurrence. OS was considered from the day of the diagnosis to death or last follow-up. Relapse and response to chemotherapy were evaluated following the response evaluation criteria in solid tumors (RECIST) (17).

Patients with FIGO stage> IIB, with missing pathological data, and those who did not provide informed consent for the enrollment in the present study were excluded.

All patients included in the analysis were divided into group 1, group 2, and group 3, based on the surgical approach as open abdominal, laparoscopic, and robotic, respectively. In addition, a comparative subanalysis between open abdominal and minimally invasive surgery (MIS) (robotic plus laparoscopic) was performed. All patients underwent RSS or fertility-sparing surgery (FSS). RSS was defined as standard staging surgery including (hysterectomy, bilateral salpingo-oophorectomy, omentectomy, systematic pelvic and para-aortic lymphadenectomy, and random peritoneal biopsies); FSS was performed in young women with IA stage disease and strong, motivated wish of conceiving, in accordance to international guidelines (4). Restaging surgery (i.e. complete lymphadenectomy, omentectomy, and possibly hysterectomy/salpingo-oophorectomy) was accomplished in all cases in which the diagnosis of ESOC was not performed intraoperatively and the malignancy was discovered only at final pathological examination.

### Statistical Analysis

Continuous variables were initially described using mean and standard deviation (SD), while categorical ones were reported as absolute numbers (%). The distribution of the variables by the surgical approach was initially assessed through a chi-squared test or analysis of variance (ANOVA) when appropriate, according to the surgical approach (open abdominal, laparoscopy, robot) and eventual relapses (yes/no). Survival analyses (i.e. overall survival and disease-free survival) were initially assessed through Kaplan Meier statistics, including overall survival/disease-free survival by eventual status (death/relapse) with and without the stratum of the surgical approach (Tarone-Ware for comparisons). Next, the exact log-rank test to standardize the follow-up medians in the survival analysis was used.

## Results

Among patients who had access to the Department of gynecology oncology at the University Hospital Fondazione Policlinico Gemelli, IRCCS during the study period, 455 met the inclusion criteria. Patients' characteristics are summarized in Table 1. Specifically, 197 (43.3%), 213 (46.8%), and 45 (9.9%) patients have been allocated to group 1 (open abdominal), group 2 (laparoscopy), and group 3 (robotic surgery), respectively, according to the surgical approach. Of the overall population of 455 patients, 316 (69.5%) subjects were diagnosed at stage I, and 139 (30.5%) at stage II. Within the group of FIGO stage II patients, 38.6% underwent open abdominal surgery while 26.3 and 15.6% of cases respectively underwent laparoscopic and robotic approaches (*p* = 0.002).

**Table 1:** Patients' characteristics.

	**Total series** **(*n*;%)** **455;100**	**Open abdominal** **(*n*;%)** **197;43.3**	**Laparoscopy** **(*n*;%)** **213; 46.8**	**Robot** **(*n*;%)** **45, 9.9**	***p* value**
**Age** (years, mean ± sd)	52.8 ± 13.1	55.4 ± 12.8	51.0 ± 13.4	50.0 ± 10.7	**0.001**
**BMI** (kg/m^2^, mean ± sd)	24.9 ± 5.8	25.5 ± 5.5	24.5 ± 5.8	24.8 ± 6.6	0.303
**ASA** status > 2	9, 5.6%	6, 9.0%	2, 2.7%	1, 5.0%	**0.030**
**FIGO Stage**					
IA	210; 46.2	78; 17.1	108; 23.7	24; 5.3	**0.042**
IB	53; 11.6	23; 11.7	25; 11.7	5; 11.1	0.993
IC	53; 11.6	20; 10.2	24; 11.3	9; 20.0	0.199
II	139; 30.5	76; 38.6	56; 26.3	7; 15.6	**0.002**
IIA	51; 11.2	30; 15.2	21; 9.9	-	**0.014**
IIB	88; 19.3	46; 23.4	35; 16.4	7; 15.6	0.174
**Histology**					
Serous carcinoma, high grade	144; 31.6	69; 35.0	59; 27.7	16; 35.6	0.248
Serous carcinoma, low grade	23; 5.1	6; 3.0%	16; 7.5	1; 2.2	0.077
Mucinous carcinoma	54; 1.9	25; 12.7	22; 10.3	7; 15.6	0.513
Clear cell carcinoma	82; 18.0	38; 19.3	38; 17.8	6; 13.3	0.651
Endometroid carcinoma	142; 31.2	53; 26.9	74; 34.7	15; 33.3	0.207
Other	10; 2.2	6; 3.0	4; 1.9	0; -	0.416
**Grading**					
G1	76; 16.9	24; 12.2	45; 21.4	7; 16.3	**0.041**
G2	94; 20.9	40; 20.3	46; 21.9	8; 18.6	0.925
G3	272; 60.4	131; 66.5	116; 55.2	25; 58.1	0.083
N/A	8; 1.8	2; 1.0	3; 1.4	3; 7.0	-
**Chemotherapy**	341, 74.9	157, 79.7	153, 71.8	31, 68.9	0.114
**Time to chemotherapy** (days; mean ± sd)	41.1 ± 14.0	41.8 ± 11.7	41.1 ± 16.6	36.4 ± 7.4	0.352

*BMI, Body Mass Index; ASA, American Society of Anesthesiologists; SD, Standard deviation. Significant values were reported in bold*.

Seventy-six patients showed a grade 1 tumor (16.9%), 95 (20.9%) a grade 2, and 272 (60.4%) a grade 3. The most frequent histotype was high-grade serous (31.6%), followed by endometrioid (31.1%), clear cells (18.0%), low-grade serous (5.1%), and mucinous (1.9%).

No difference in adjuvant chemotherapy (79.7 vs. 71.8 vs. 68.9%, in group 1, 2, and 3, respectively, *p* = 0.114) or mean time to chemotherapy (41.8 days ± 11.7 vs. 41.1 days ± 16.6 vs. 36.4 days ± 7.4, in group 1, 2, and 3, respectively, *p* = 0.352) was observed in the three different groups.

The median age at diagnosis was 52.8 years. Patients in the open abdominal group showed a higher mean age (55.4 years) than in the laparoscopic (51.0) and robotic (50.0) groups. Most of FIGO stage IA patients were clustered in group 2 (108 cases, *p* = 0.042), while FIGO stage II patients were more represented in group 1 (76 cases, *p* = 0.002).

### Surgical Outcomes

Ninety-seven (21.3%) patients underwent FSS, 358 (78.7%) underwent RSS, and 171 (37.6%) were subjected to restaging surgery after accidental OC diagnosis during previous salpingo-oophorectomy or cystectomy.

As shown in Table 2, no difference in intraoperative complications was recorded in the three groups (*p* = 0.709); conversely, a significant difference occurred in postoperative complications (16.2% in group 1 vs. 3.8% group 2 vs. 11.1% group 3, *p* = 0.004). In particular, postoperative anemia (9 cases vs. 0 vs. 0, *p* = 0.002), and abdominal effusion (5 cases vs. 0 vs. 0, *p* = 0.036), occurred more often in the open abdominal group, while lymphocele (3 cases vs. 0 vs. 2, *p* = 0.026) was more frequent in the robotic one. Finally, grade 1/2 Claiven-Dindo complications were more often reported in group 1 than in group 2 or 3 (28 cases vs. 8 cases vs. 5 cases, *p* = 0.008). Complications according to Claiven-Dindo classification in the different surgical approaches are shown in Table 3. No statistically significant difference compared to the other groups (*p* = 0.112) was observed, but four (2%) patients required reintervention for postoperative bowel perforation in the open abdominal group. Furthermore, these differences remained even when grouping laparoscopic and robotic patients into the MIS vs. the laparotomic approach.

**Table 2:** Intraoperative and postoperative complications.

	**Total (n;%)** **455;100**	**Open abdominal** **(n;%)** **197;43.3**	**Laparoscopy (n;%)** **213; 46.8**	**Robot (n;%)** **45, 9.9**	***p* value**	**MIS (*n*;%)** **258, 56.7**	***p* value** **LPT vs. MIS**
**Intraoperative**	8;1.8	5;2.5	2;0.9	1; 2.2	0.709	3; 1.2	0.338
Pleural effusion	0;-	0;-	0;-	0;-	-	-	-
Pulmonary embolism	0;-	0;-	0;-	0;-	-	-	-
Hemorrhage	0;-	0;-	0;-	0;-	-	-	-
Vascular lesions	2; 0.4	1; 0.5	1; 0.5	0; -	0.894	1; 0.4	0.679
Ureteral lesions	4; 0.9	2; 1.0	1; 0.5	1; 2.2	0.501	2; 0.8	0.581
Intestinal lesions	2; 0.4	2; 1.0	0; -	0; -	0.621	-	0.268
Laparotomic conversions	-	-	8; 3.8	1; 2.2	0.358	9; 3.5	-
**Postoperative**	45;8.9	32;16.2	8;3.8	5;11.1	**0.004**	**13; 5.0**	**0.022**
Ureteral lesions	4; 0.9	2; 1.0	1; 0.5	1; 2.2	0.501	2; 0.8	0.723
Intestinal lesions	2; 0.4	2; 1.0	0; -	0; -	0.621		
Pleural effusion	1; 0.2	1; 0.5	0; -	0; -	0.519	0;-	0.433
Pulmonary embolism	1; 0.2	1; 0.5	0; -	0; -	0.519	0;-	0.433
Hemorrhage	1; 0.2	0; -	1; 0.5	0; -	0.566	1; 0.4	0.567
Pneumonia	3;0.7	2; 1.0	0; -	1; 2.2	0.176	1; 0.4	0.400
Sepsis	7;1.5	5;2.5	2;0.9	0; -	0.285	2; 0.8	0.130
Anemia	9	9; 4.6	0; -	0; -	**0.002**	**0;-**	**<0.001**
Abdominal effusion	5	5;2.5	0; -	0; -	**0.036**	**0;-**	**0.015**
Urinary tract infections	1; 0.2	0; -	1; 0.5	0; -	0.566	1; 0.4	0.567
Intestinal Pseudo-Obstruction	3;0.7	0; -	2;0.9	1; 2.2	0.198	3; 1.2	0.181
Lymphocele	5;1.1	3;1.5	0; -	2;4.4	**0.026**	2; 0.8	0.375
Fistula	1; 0.2	0; -	1; 0.5	0; -	0.566	1; 0.4	0.567
Wound infection	2; 0.4	2; 1.0	0; -	0; -	0.268	0; -	0.187

*LPS, Laparoscopy; LPT, Laparotomy; MIS, Minimally invasive. Significant values were reported in bold*.

**Table 3:** Complications according to Claiven-Dindo classification in the different kinds of surgeries.

**General population**	**Total**	**Open abdominal**	**Laparoscopy**	**Robot**	***p* value**	**MIS**	***p* value (LPT vs. MIS)**
G1-G2 *	41	28	8	5	***p** **=*** **0.008**	**13**	**<0.001**
G3-G4**	4	4	0	0	*p =* 0.112	**0**	**0.035**
**Fertility sparing**							
G1-G2	6	2	2	2	*p =* 0.318	4	0.264
G3-G4**	1	2	0	0	*p =* 0.139	0	0.607
**Radical surgical staging**							
G1-G2	35	26	6	3	*p =* 0.076	9	0.190
G3-G4**	3	2	1	0	*p =* 0.156	1	0.072

* *Vascular lesions, ureteral lesions, pulmonary embolism, pneumonia, sepsis, anemia, urinary tract infection, ileus, lymphocele, fistula, surgical site infection*.

** *Intestinal lesions requiring reintervention. LPS, Laparoscopy; LPT, Laparotomy; MIS, Minimally invasive. Significant values were reported in bold*.

As shown in Table 4, a higher estimated blood loss (EBL) (274.5 vs. 142.2 vs. 79.3 ml, *p* < 0.001), longer hospital stay (5.8 vs. 2.6 vs. 2.8 days, *p* < 0.001), and longer operative time (243.0 vs. 224.1 vs. 197.2 min, *p* = 0.004) were recorded in group 1 vs. group 2 vs. group 3, respectively. Conversely, these differences were nullified by pooling MIS patients vs. the laparotomic group (Table 4).

**Table 4:** Surgical outcomes.

	**Total (*n*;%)** **455;100**	**Open abdominal** **(*n*;%)** **197;43.3**	**Laparoscopy** **(*n*;%)** **213; 46.8**	**Robot** **(*n*;%)** **45, 9.9**	***p* value**	**MIS** **(n;%)**	***p* value** **LPT vs. LPS**
**Type of surgery**							
Fertility sparing	97; 21.3	19; 9.6	62; 29.1	16; 35.6	**<0.001**	78; 17.1	**<0.001**
Radical surgical staging	358; 78.7	178; 39.1	151; 33.2	29; 6.4	**<0.001**	**180; 39.6**	**<0.001**
Restaging	171; 37.6	19; 0.2	110; 24.2	42; 9.2	**<0.001**	**152; 33.4**	**0.374**
**Aortic lymph nodes removed** Number (mean ± sd)	9 ± 8.4	8 ± 9.9	8.5 ± 7.3	9 ± 6.9	0.197	10.8 ± 7.2	0.735
**Pelvic lymph nodes removed** Number (mean ± sd)	10 ± 8.0	10 ± 8.3	9 ± 7.5	12.5 ± 8.6	0.195	12.7 ± 9.1	0.930
**Estimated blood loss** (mL; mean ± sd)	179.0 ± 209.8	274.5 ± 229.3	142.2 ± 201.6	79.3 ± 47.3	**<0.001**	127.3 ±178.5	0.632
**Operative time** (minutes; mean ± sd)	227.5 ± 81.7	243.0 ± 83.6	224.1 ± 78.9	197.2 ± 79.4	**0.004**	245 ± 79.1	0.842
**Hospital stay** (days; mean ± sd)	3.9 ± 5.3	5.8 ± 7.8	2.6 ± 1.1	2.8 ± 1.7	**<0.001**	2.6± 1.2	0.610

*LPS, Laparoscopy; LPT, Laparotomy; MIS, Minimally invasive. Significant values were reported in bold*.

Finally, no significant difference in the number of pelvic (*p* = 0.197) and lumboortic (*p* = 0.195) lymph nodes removed was observed among the three groups.

### Survival Analysis

Twenty total deaths occurred in the entire population, 12 (2.6%) in group 1, 6 (1.3%) in group 2, and 2 (0.4%) in group 3 (*p* = 0.267). Sixty total relapses (13.2%) were found in the whole series; of them, 54 occurred in patients who underwent RSS, and 24 were patients at FIGO stage II.

**Figure 1:**
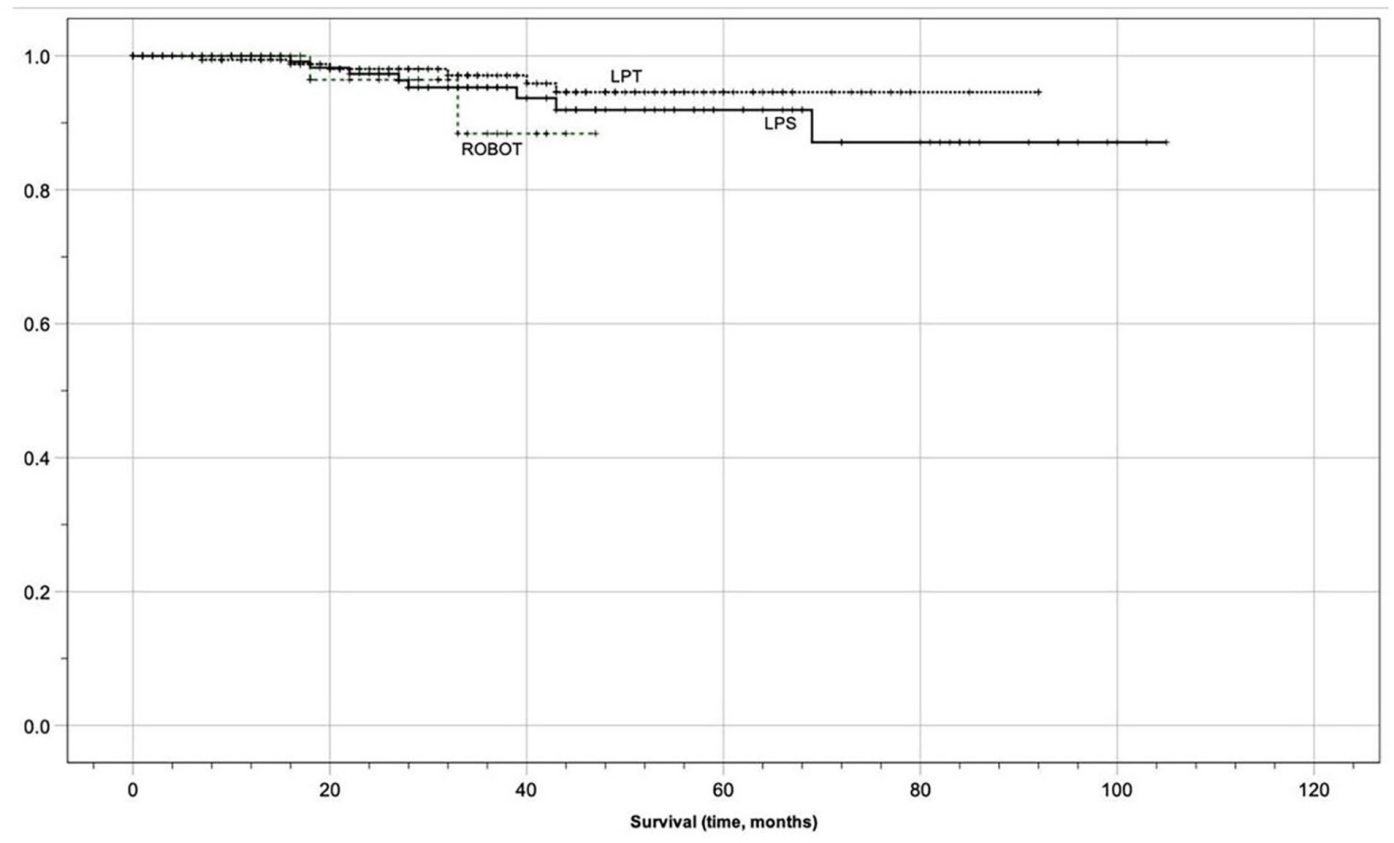
Kaplan meier survival analysis (overall survival).

**Figure 2:**
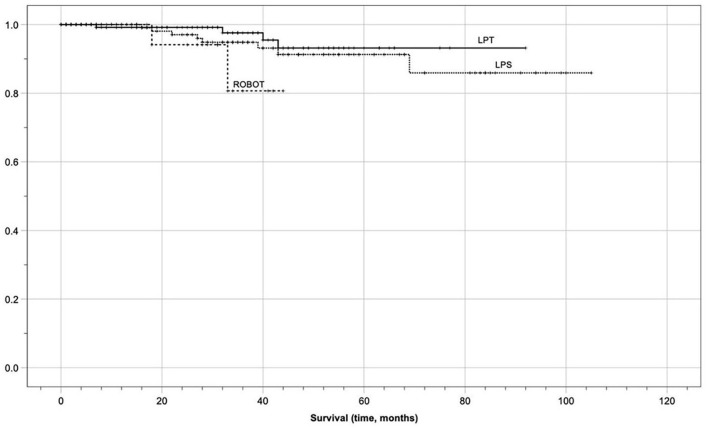
Kaplan meier survival analysis (disease-free survival).

In the entire population, after applying the exact log-rank test, to smooth out the follow-up discrepancies, no statistically significant differences in median OS (32 months in group 1 vs. 31 in group 2 vs. 25 in group 3, *p* = 0.481) and DFS (26 months in group 1 vs. 29 in group 2 vs. 24 in group 3, *p* = 0.178) were found in the three groups. Oncological outcomes are displayed in Table 5 and Kaplan-Meier analysis after the exact log-rank test is shown in Figures 1, 2. Furthermore, these differences remained non-statistically significant even when comparing MIS with laparotomic group (DFS 28 vs. 26 months, *p* = 0.067, and, OS 29 vs. 32 months, *p* = 0.441, respectively). Kaplan Meier analysis showed 1-year OS of 100 vs. 100%, 3-years OS of 100 vs. 100%, and 5-years OS of 99.2 vs. 99.5% in the laparotomic vs. MIS approach (95% Confidence Interval 26.37–31.75).

**Table 5:** Survival analysis.

	**Total median (range)** **455;100%**	**Open abdominal median** **(range)** **197;43.3%**	**Laparoscopy median (range)** **213; 46.8%**	**Robot median (range)** **45, 9.9%**	***p* value**	**MIS**	***p* value** **(LPT vs. MIS)**
**General population**							
DFS (months)	28 (10–44)	26 (8–43.5)	29 (10.8–48)	24 (12–31.5)	0.178	28 (10.8–31.5)	0.067
OS (months)	30 (12–47)	32 (11.5–52.5)	31 (13–48)	25 (12–33)	0.481	29 (12–33)	0.441
Relapse (*n*; %)	60; 13.2	39; 19.8	19; 8.9	2; 4.4	0.072	21; 8.1	0.064

*DFS, Disease-Free Survival; OS, Overall survival; LPS, Laparoscopy; LPT, Laparotomy; MIS, Minimally invasive*.

As reported in Table 6, at univariate analysis FIGO stage I patients (*p* = 0.004) showed lower recurrence rates when compared to FIGO stage II patients. Furthermore, the subanalysis of patients undergoing complete surgical staging showed a higher relapse rate in the laparotomic than the MIS group, 68.5 vs. 31.5% (*p* = 0.002).

**Table 6:** Univariate analysis.

**Patients underwent complete surgical staging**	**Recurrence** **54** **number %**		**No recurrence** **304** **number %**		***p* value**
Age > 50 years	36	66.7%	200	65.8%	0.9
BMI > 30 kg/m^2^	7	13.0%	36	11.8%	0.815
ASA > 2	2	3.7%	7	2.3%	0.544
**Hystotype**					0.178
Serous. high grade	24	44.4%	105	34.5%	
Serous. low grade	1	1.9%	16	5.3%	
Mucinous	3	5.6%	33	10.9%	
Clear cells	14	25.9%	50	16.4%	
Endometrioid	11	20.4%	92	30.3%	
Others	1	1.9%	8	2.6%	
**Grading**					0.053
G1	4	7.4%	48	15.8%	
G2	6	11.1%	62	20.4%	
G3	44	81.5%	188	61.8%	
**FIGO stage**					0.156
I	13	24.1%	115	37.8%	
IA	2	3.7%	24	7.9%	
IB	6	11.1%	34	11.2%	
IC	9	16.7%	38	12.5%	
IIA	7	13.0%	37	12.2%	
IIB	17	31.5%	56	18.4%	
II vs. I	24	44.4%	93	30.6%	**0.046**
**Surgical approach**					**0.002**
Laparotomy	37	68.5%	141	46.4%	
MIS	17	31.5%	163	53.6%	
Introperative complication	3	5.6%	5	1.6%	0.073
Postoperative complication	3	5.6%	30	9.9%	0.293
Chemiotherapy	47	87.0%	227	74.7%	0.072

*G, Grading; LPT, Laparotomy; LPS, Laparoscopy; ASA, American Society of Anesthesiologists; MIS, Minimally invasive surgery. Significant values were reported in bold*.

## Discussion

The present study showed that minimally invasive comprehensive surgical staging for ESOC was safe and associated with a lower rate of postoperative morbidity, compared to the traditional open abdominal approach. Furthermore, there was no statistically significant difference in patients' survival among the laparotomic, laparoscopic, and robotic groups.

In line with our results, Magrina et al. (14) reported an overlapping OS between these three different surgical approaches in a series of both early and advanced epithelial ovarian cancer. In the sub-analysis of early-stage cases, the same authors reported superimposable results in terms of oncological outcomes with fewer surgical complications in the MIS group (laparoscopic and robotic) when compared to the traditional open abdominal surgery. Several authors confirmed these findings: in particular, Liu et al. (18), analyzing the most relevant studies in the literature, demonstrated a comparable survival between the minimally invasive and the open approach in both early and advanced FIGO stage (19–21).

An important hurdle we faced to obtain a meaningful survival analysis was related to the wide heterogeneity of the population enrolled. As recently reported by Shi et al. (13), the heterogeneity of OC population in the different trials can afflict survival outcomes making comparisons unreliable. Furthermore, Falcetta et al. (22) stressed that trouble for data analysis of ESOC patients was related to the variety of the treatments proposed, ranging from fertility-sparing to radical surgery. Trying to overcome these limitations, we enrolled a large single-center series and we focused the survival analysis on the comprehensive surgically staged patients. Furthermore, an exact log-rank test for survival analysis was used to reduce the consequent bias and to adequately match the patients.

It is well established that the main prognostic factor affecting OC recurrence is the FIGO stage (23). In our series, group 1 showed a greater proportion of FIGO stage II cases (*p* = 0.014) than the two minimally invasive groups and, as expected, the relapse rate appeared higher in this group when compared to the other approaches. However, after standardization of follow-up with exact log-rank test, no significant difference was found in DFS within the three approaches (*p* = 0.178). In line with our results, Zhang et al. (24), in a meta-analysis including 8 studies, reported no significant difference in DFS of ESOC patients subjected to laparotomy vs. laparoscopy with fewer complication rates and shorter hospital stay in the latter group.

In contrast with previously reported studies, we found that patients undergoing robotic surgery had a shorter operative time (8, 25). This finding may be due to the very precise three-dimensional movement of the robotic arms that could be useful and time-saving, especially when facing complex procedures such as lumboortic lymphadenectomy (26). Furthermore, the greater number of complete surgical staging procedures in the open abdominal and laparoscopic groups compared to the robotic one could justify this result.

Compared to previous retrospective studies and clinical trials, our series showed a higher rate of mild complications (G1-G2 according to Claiven Dindo classification) in the open abdominal group (27, 28). In addition, these differences in G1-G2 complications not only remained when comparing the MIS group with the laparotomic approach but also included the G3-G4 complications. This finding could be related to the mean age (*p* = 0.001), ASA status (*p* = 0.030), and FIGO stage II (*p* = 0.002) which were higher in group 1. As reported by Patankar et al., all these demographic factors are associated with worse surgical outcomes (29).

We know that this study has several possible limitations due to its retrospective nature. Given the rarity and the good prognosis of ESOC, only 20 death events were observed in the entire population, and this may have influenced the survival analysis.

Furthermore, the different complications rate reported could be influenced by the heterogeneity of the interventions performed in the three groups. Therefore, prospective studies with standardization of interventions performed in the various approaches are needed to confirm our results.

On the other hand, we emphasize that this study has important strengths such as the size of the sample analyzed, the long follow-up time, the close selection of the patients analyzed, and the single oncological tertiary center experience reported.

## Conclusions

After follow-up standardization, we observed no statistically significant difference in OS and DFS among the three groups analyzed (open abdominal, laparoscopic, and robotic).

The open abdominal approach in ESOC was associated with a higher mild complication rate than the laparoscopic and robotic ones. Based on these findings, the minimally invasive approach should be preferred in selected patients and in tertiary cancer centers.

## Data Availability Statement

The raw data supporting the conclusions of this article will be made available by the authors, without undue reservation.

## Ethics Statement

The studies involving human participants were reviewed and approved by Dipartimento Scienze della Salute della Donna e del Bambino, Fondazione Policlinico Universitario A. Gemelli IRCCS, Roma, Italy. IRB code n. CICOG-31-10-18/212. The patients/participants provided their written informed consent to participate in this study.

## Author Contributions

SC, VC, and AR: conceptualization, methodology, and writing-original draft preparation. MR and VR: software and data curation. SU, GC, and SG: visualization and investigation. AF, GS, and FC: supervision, validation, reviewing, and editing. All authors contributed to the article and approved the submitted version.

## Conflict of Interest

The authors declare that the research was conducted in the absence of any commercial or financial relationships that could be construed as a potential conflict of interest.

## Publisher's Note

All claims expressed in this article are solely those of the authors and do not necessarily represent those of their affiliated organizations, or those of the publisher, the editors and the reviewers. Any product that may be evaluated in this article, or claim that may be made by its manufacturer, is not guaranteed or endorsed by the publisher.
